# Upgrading dilute ethanol to odd-chain carboxylic acids by a synthetic co-culture of *Anaerotignum neopropionicum* and *Clostridium kluyveri*

**DOI:** 10.1186/s13068-023-02336-w

**Published:** 2023-05-17

**Authors:** Ivette Parera Olm, Diana Z. Sousa

**Affiliations:** 1 Laboratory of Microbiology, Wageningen University & Research, Wageningen, The Netherlands; 2 Centre for Living Technologies, Eindhoven-Wageningen-Utrecht Alliance, Utrecht, The Netherlands

**Keywords:** Chain elongation, Ethanol, Propionate, Valerate, Co-cultivation, *Clostridium kluyveri*, *Anaerotignum neopropionicum*

## Abstract

**Background:**

Dilute ethanol streams generated during fermentation of biomass or syngas can be used as feedstocks for the production of higher value products. In this study, we describe a novel synthetic microbial co-culture that can effectively upgrade dilute ethanol streams to odd-chain carboxylic acids (OCCAs), specifically valerate and heptanoate. The co-culture consists of two strict anaerobic microorganisms: *Anaerotignum neopropionicum*, a propionigenic bacterium that ferments ethanol, and *Clostridium kluyveri*, well-known for its chain-elongating metabolism. In this co-culture, *A. neopropionicum* grows on ethanol and CO_2_ producing propionate and acetate, which are then utilised by *C. kluyveri* for chain elongation with ethanol as the electron donor.

**Results:**

A co-culture of *A. neopropionicum* and *C. kluyveri* was established in serum bottles with 50 mM ethanol, leading to the production of valerate (5.4 ± 0.1 mM) as main product of ethanol-driven chain elongation. In a continuous bioreactor supplied with 3.1 g ethanol L^−1^ d^−1^, the co-culture exhibited high ethanol conversion (96.6%) and produced 25% (mol/mol) valerate, with a steady-state concentration of 8.5 mM and a rate of 5.7 mmol L^−1^ d^−1^. In addition, up to 6.5 mM heptanoate was produced at a rate of 2.9 mmol L^−1^ d^−1^. Batch experiments were also conducted to study the individual growth of the two strains on ethanol. *A. neopropionicum* showed the highest growth rate when cultured with 50 mM ethanol (*μ*_max_ = 0.103 ± 0.003 h^−1^) and tolerated ethanol concentrations of up to 300 mM. Cultivation experiments with *C. kluyveri* showed that propionate and acetate were used simultaneously for chain elongation. However, growth on propionate alone (50 mM and 100 mM) led to a 1.8-fold reduction in growth rate compared to growth on acetate. Our results also revealed sub-optimal substrate use by *C. kluyveri* during odd-chain elongation, where excessive ethanol was oxidised to acetate.

**Conclusions:**

This study highlights the potential of synthetic co-cultivation in chain elongation processes to target the production of OCCAs. Furthermore, our findings shed light on to the metabolism of odd-chain elongation by *C. kluyveri*.

**Supplementary Information:**

The online version contains supplementary material available at 10.1186/s13068-023-02336-w.

## Background

Sustainable chemical production is a key factor in reducing our strong dependency on oil and in the mitigation of climate change [1, 2]. In the last century, numerous microbial processes have been developed and deployed for the production of biochemicals and biofuels [e.g., lactic acid fermentation, acetone–butanol–ethanol (ABE) fermentation], establishing the grounds for a biobased economy [3]. Traditionally, these microbial processes rely on the fermentation of sugars, either from crops including corn or sugarcane, or from hydrolysed lignocellulosic biomass. While these are feedstocks available in high quantities, the former pose ethical concerns as they compete with food and feed applications. The latter, on the other hand, require costly pre-treatments and contain a significant fraction of recalcitrant lignin (15–25% of total dry matter). To circumvent these limitations, production platforms based on the use of non-conventional feedstocks (e.g., CO_2_, glycerol, organic waste streams) are expected to become more prominent in the chemical industry [4, 5]. In the last decade, significant advances have been made on the development of a C1-based biorefinery [6]. C1 feedstocks comprise formate, methanol, CO_2_ and CO. A mixture of CO, H_2_ and CO_2_, known as synthesis gas (syngas), can be obtained via gasification of hydrocarbon resources, including organic wastes or lignocellulosic biomass [7]. In addition, syngas-like streams are generated at energy-intensive industrial sites (e.g., steel mills) [8], and syngas production by CO_2_–water electrolysis using renewable energy is becoming increasingly feasible [9–11]. Because syngas composition varies depending on the material and technology used for its generation, this feedstock is particularly suited for microbial fermentation, which is more tolerant than chemical conversion (i.e., Fischer–Tropsch) to varying CO:H_2_ ratios and gas impurities [12–14].

Syngas can be metabolised by acetogens: strict anaerobic bacteria that use the Wood–Ljundahl pathway for CO_2_ fixation. Acetogens can use reducing equivalents from H_2_, CO or other substrates, and produce acetate and ethanol as main products [15]. So far, syngas fermentation has been industrially deployed almost exclusively for the production of ethanol; yet, to maximise its value as sustainable production platform, it is essential to expand the array of products [16, 17]. Since syngas fermentation effluent contains a mixture of ethanol and acetate, one logical approach for its upgrading is the integration with the chain-elongation platform. Chain elongation is the anaerobic process in which short-chain carboxylic acids (SCCAs; acetate, propionate, butyrate) are converted to medium-chain carboxylic acids (MCCAs; valerate, caproate) provided that an electron donor (e.g., ethanol, lactate) is supplied [18–20]. This conversion is the result of the reverse β-oxidation pathway, a cycle that elongates a carboxylic acid two carbons at a time by adding an acetyl-CoA derived from the electron donor. *Clostridium kluyveri* was the first isolated bacterium performing chain elongation, and since then it has been thoroughly characterised [21–24]. In recent years, several studies have demonstrated the feasibility of coupling syngas fermentation to chain elongation for the production of MCCAs and higher alcohols [25]. One adopted strategy is the one-pot conversion of CO/syngas by open mixed cultures traditionally used in anaerobic digestion, which can be enriched for chain-elongating microorganisms [26–33]. Despite their robustness and suitability for waste-fed processes, these cultures require rather long acclimation and fermentation times when applied to syngas fermentation, and the abundance of competing pathways (e.g., methanogenesis) compromises product selectivity. A popular alternative is the use of synthetic co-cultures of acetogens with *C. kluyveri* [34–37], which are based on CO scavenging by the acetogen and direct cross-feeding of ethanol and acetate. Two-stage processes, where syngas fermentation and chain elongation take place in separate bioreactors, have also been proposed [38–41], with the advantage that the operational conditions can be optimised for each conversion.

Even-chain MCCAs have received the most attention as target products, largely because intermediates with even number of carbon (i.e., acetate, ethanol, butyrate) are more commonly found in syngas fermentation effluent and in acidogenic waste streams than odd-numbered carbon constituents [42]. Caproate (C6) and caprylate (C8) have attracted special interest due to their higher economic value and easiness to extract from the water broth compared to butyrate (C4). However, to avert market saturation, interest has grown in diversifying away from the caproate platform towards other products, such as branched carboxylates and odd-chain carboxylic acids (OCCAs) [43–45]. In particular, OCCAs [e.g., valerate (C5) and heptanoate (C7)] are valuable building blocks with growing demand from the chemical and cosmetics industries [46]. To date, only a few studies have addressed the production of OCCAs in ethanol-based chain elongation systems; in these cases, the most common approach has been the supplementation of propionate in mixed cultures [47–50]. In addition, the physiology of odd-chain elongation in *C. kluyveri* has been considerably less well-studied than the even-chain metabolism, with exception of early studies [21, 23] and a recent investigation by Candry and co-authors [51].

In this work, we propose an alternative to mixed cultures to target the production of OCCAs via ethanol-based chain elongation: a synthetic co-culture of *C. kluyveri* with the propionigenic bacterium *Anaerotignum neopropionicum* (formerly, *Clostridium neopropionicum* [52]). *A. neopropionicum* is among the few propionigenic bacteria described to date with the ability to ferment ethanol while fixing CO_2_ [53, 54]. Based on the physiology of the microorganisms, it is anticipated that a co-culture of *A. neopropionicum* and *C. kluyveri* supplied with ethanol and CO_2_ will produce valerate and heptanoate, with propionate as intermediate. Acetate is produced in this system; therefore, even-chain products are also expected. We established the *A. neopropionicum*–*C. kluyveri* co-culture in serum bottles and tested its productivity in an ethanol-fed chemostat bioreactor at increasing ethanol loading rates (ELRs). We also performed pure culture experiments in serum bottles to gain insight into the metabolism of ethanol fermentation in *A. neopropionicum* and the use of propionate during chain elongation by *C. kluyveri*. Ultimately, our goal was to evaluate the feasibility of applying this co-culture to upgrade syngas fermentation effluent to OCCAs.

## Methods

### Microbial strains and cultivation medium

*C. kluyveri* DSM 555^T^ and *A. neopropionicum* DSM 3847^T^ were obtained from the German Collection of Microorganisms and Cell Cultures (Leibniz Institute DSMZ—German Collection of Microorganisms and Cell Cultures GmbH, Braunschweig, Germany). Both strains were cultivated anaerobically in medium containing (per litre): 0.9 g NH_4_Cl, 0.8 KCl, 0.3 g NaCl, 0.2 g KH_2_PO_4_, 0.4 g K_2_HPO_4_, 0.2 g MgSO_4_·7 H_2_O, 0.04 g CaCl_2_·2 H_2_O, 3.0 g NaHCO_3_, 0.5 g yeast extract and 10 mL trace element solution from DSMZ medium 318. The pH was adjusted to 7. Anaerobic bottles of 120 mL were filled with 50 mL medium and a N_2_/CO_2_ headspace (80:20% v/v; 170 kPa). For culture maintenance, *C. kluyveri* was grown on 90 mM ethanol and 75 mM acetate, incubated statically at 37 °C. *A. neopropionicum* was grown on 50 mM ethanol, statically at 30 °C.

### Batch experiments in serum bottles

A series of batch experiments were carried out in serum bottles and medium as described above. Ethanol, acetate and propionate were added in a concentration of 25–1000 mM depending on the experiment, as detailed in the Results section. The headspace of the bottles was filled with N_2_/CO_2_ (80:20% v/v; 170 kPa). Bottles were inoculated with 2% (v/v) of exponentially growing cultures of *C. kluyveri* and/or *A. neopropionicum*. For monoculture experiments of *C. kluyveri* with propionate in the medium, pre-cultures used for inoculation were transferred at least three times in medium containing propionate. Experiments with monocultures of *A. neopropionicum* and of *C. kluyveri* were incubated at 30 °C and 37 °C, respectively. Co-cultures of *A. neopropionicum* with *C. kluyveri* were incubated at 35 °C. All bottle experiments were done in triplicates. Liquid samples (1 mL) were routinely taken for analyses of alcohols and carboxylic acids, cell density and pH. Gas samples (0.2 mL) were routinely taken for analysis of headspace composition (H_2_, CO_2_). For each experiment, cell dry weight (CDW), yields, carbon and electron balances were determined from an additional, equivalent set of bottles that was only sampled at the start and end of cultivation.

### Bioreactor setup

An ethanol-limited chemostat experiment was carried out to test the performance of the co-culture of *A. neopropionicum* and *C. kluyveri* at increasing ethanol loading rates (ELRs). A 1.3 L bioreactor vessel (DASGIP® Bioblock, Eppendorf, Germany) with a working volume of 0.7 L was operated anaerobically in continuous mode. The composition of the medium was as described above, except that NaHCO_3_ was omitted during continuous operation. The reactor was equipped with pH, redox and temperature sensors. The system was operated at 35 °C and pH 7, the latter controlled by the addition of 3 M KHCO_3_. Agitation was set at 200 rpm. Mass flow controllers (Dasgip MX4/4, Eppendorf, Germany) regulated the inflow of gas (N_2_ or N_2_/CO_2_), which was supplied aseptically via a 0.2 µm filter. Liquid samples were routinely taken for analyses of ethanol and carboxylic acids, cell density and CDW. Gas samples of the headspace were taken for determination of gas composition. Gas and liquid outflow rates were regularly measured during operation. Phase-contrast microscopy (Axio Scope A1, Zeiss) was used periodically to inspect the co-culture.

### Bioreactor operation

The co-culture of *A. neopropionicum*–*C. kluyveri* was cultivated in the continuous stirred-tank reactor (CSTR) operated without interruption for 160 days. The experiment can be divided in seven phases, A–G, preceded by a brief batch phase; the operating conditions of each phase are detailed in Table 1. Start-up of the bioreactor was done as follows: the autoclaved reactor vessel, containing only mineral medium, trace elements and resazurin, was connected to the system and flushed with N_2_ (5 L h^−1^) for ~ 3 h to establish anaerobic conditions. Next, the gas inflow was switched to N_2_/CO_2_ (80:20% v/v) and the flowrate adjusted to 1.8 L h^−1^ (0.04 vvm). The following supplements were then added aseptically to the medium from anaerobic, sterile stock solutions: yeast extract, vitamins, NaHCO_3_ and L-cysteine-HCl, in the concentrations given above. 50 mM ethanol was supplemented as substrate. When the redox potential dropped below -300 mV, the bioreactor was inoculated with 40 mL (~ 6% v/v) of exponentially growing pure cultures of *A. neopropionicum* and *C. kluyveri*. The co-culture was grown in batch for 50 h, when ethanol became depleted. At this point, the continuous operation was initiated. Fresh medium containing ethanol (initially, 50 mM) was supplied aseptically from a 20-L tank via a peristaltic pump (Masterflex, Germany). The medium tank was flushed with N_2_ aseptically thorough the whole operation to ensure anaerobic conditions. The hydraulic retention time (HRT) was initially set at 36 h (dilution rate, D = 0.028 h^−1^), resulting in an ELR of 1.7 g ethanol L^−1^ d^−1^ (phase A). Each subsequent phase was characterised by an increase of the ELR or by the adjustment of a reactor parameter. Modification of the ELR was done either by changing the medium inflow rate (and, thus, the HRT) or by increasing the ethanol concentration in the medium tank (Table 1).

**Table 1:** Operating conditions of the bioreactor

Phase	Period (days)			ELR (g L^−1^ d^−1^) [mM d^−1^]	HRT (h)	Ethanol inflow (mM)	Remark
Batch	0	–	2.1	–	–	–	–
A	2.1	–	3	1.7 [35]	36	51	Start of the continuous operation
B	3	–	17.8	1.7 [35]	36	51	Gas flowrate increased to 0.07 vvm
C	17.8	–	29.7	3.1 [64]	36	95	–
D	29.7	–	32.9	8.0 [171]	42	300	–
E.1	32.9	–	43.7	6.3 [122]	54	300	–
E.2	43.7	–	100.7	6.3 [122]	54	300	Technical issues^a^
F	100.7	–	155.7	6.7 [145]	54	319	Technical issues fixed and new medium tank
G	155.7	–	160	6.7 [145]	54	320	pH increased to 7.3

*ELR* ethanol loading rate

*HRT* hydraulic retention time

^a^The outflow line clogged on day 55. In addition, during this period ethanol was slowly being stripped out of the medium tank

### Analytical techniques

Gaseous compounds (CO_2_, H_2_) were analysed in a gas chromatograph (Compact GC 4.0, Global Analyser Solutions, The Netherlands) equipped with two channels and a thermal conductivity detector. H_2_ was detected using a Molsieve 5A column operated at 100 °C and coupled to a Carboxen 1010 pre-column. Determination of CO_2_ was done in a Rt-Q-BOND column operated at 60 °C. In both channels, argon was used as carrier gas. Concentrations of soluble compounds, namely, ethanol, propanol, lactate and C2–C7 carboxylic acids, were determined by high-performance liquid chromatography (HPLC; LC-2030C, Shimadzu, Japan). The apparatus was equipped with a Shodex SH1821 column operating at 55 °C. 0.01 N H_2_SO_4_ was used as eluent and the flowrate set at 1 mL min^−1^. Amounts detected in a concentration of < 0.3 mM could not be accurately quantified and are considered traces. The Chromeleon™ data analysis software (Thermo Fisher Scientific), version 7.2.9, was used for both GC and HPLC peak integration and analysis.

Microbial growth was estimated based on the measurement of optical density at 600 nm (OD_600_) using a spectrophotometer (UV-1800, Shimadzu, Japan). CDW was determined by gravimetric analysis: pellets from a known culture volume (~ 50 mL) were washed twice in deionised water, resuspended and transferred into pre-weighed aluminium trays. These were dried overnight at 105 °C and weighed again the day after.

### Calculations

Specific growth rates (*μ*, expressed in h^−1^) in batch incubations were determined as the slope of the linear regression (three to five points; *R*^2^ ≥ 0.99) derived from the integrated mass balance of cells:

$${\text{ln}}\left( {C_{X,t} } \right){ = }{\mkern 1mu} \mu {\mkern 1mu} \cdot t{\text{ + ln(}}C_{{X,t_{0} }} {)}$$

where *C*_*X*_ is the cell concentration as OD_600_ during the exponential phase (*t*_0_–*t*).

In batch experiments of *A. neopropionicum*, substrate (ethanol) uptake rates (*q*_*S*_) and production rates of propionate (*q*_*P*_) and acetate (*q*_*A*_) (defined collectively as q-rates) were calculated from the respective integrated mass balances which, resolved in combination with the biomass mass balance, give:

$$N_{S} \left( t \right) - {\mkern 1mu} N_{S} \left( {t_{0} } \right){\mkern 1mu} { = }{\mkern 1mu} \frac{{q_{S \, } }}{\mu }{\mkern 1mu} \cdot \left( {N_{X} \left( t \right) - {\mkern 1mu} N_{X} \left( {t_{0} } \right)} \right)$$

$$N_{P} \left( t \right) - {\mkern 1mu} N_{P} \left( {t_{0} } \right){\mkern 1mu} { = }{\mkern 1mu} \frac{{q_{P \, } }}{\mu }{\mkern 1mu} \cdot {\mkern 1mu} \left( {N_{X} \left( t \right) - {\mkern 1mu} N_{X} \left( {t_{0} } \right)} \right)$$

$$N_{A} \left( t \right) - {\mkern 1mu} N_{A} \left( {t_{0} } \right){\mkern 1mu} { = }{\mkern 1mu} \frac{{q_{A \, } }}{\mu } \cdot {\mkern 1mu} \left( {N_{X} \left( t \right) - {\mkern 1mu} N_{X} \left( {t_{0} } \right)} \right)$$

where q-rates are calculated from the slope of the linear regression (*R*^2^ ≥ 0.99) with μ known (calculated as described in the lines above). *N*_*S*_, *N*_*P*_ and *N*_*A*_ are measured amounts (mmol) of ethanol, propionate and acetate, respectively, during the exponential phase (t_0_–t). N_X_ (mg CDW) at corresponding timepoints were indirectly determined from OD_600_ values using the following relationship, that we determined experimentally for *A. neopropionicum* growing on ethanol:

$${\text{CDW}} ({\text{mg L}}^{-1}) \, = \, \frac{\left({\text{OD}}_{600} - 0.016 \right)}{0.0032}$$

q-rates are given in mmol g CDW^−1^ h^−1^.

In batch cultivations, biomass and product yields were calculated as follows:

$${\text{Biomass yield (}{{Y}}}_{{X}}\text{)} \,= \, \frac{\text{g CDW formed}}{\text{mol ethanol consumed}}$$

$${\text{Product yield (}{{Y}}}_{{i}}\text{) = }\frac{\text{mol product}\,{i}\,{\text{formed}}}{\text{mol ethanol consumed}}$$

Product specificity (mol/mol) and selectivity (mol e^−^ eq./mol e^−^ eq.) were calculated as follows:

$$\text{Specificity of product}\,{i}\,{(\%)}= \frac{\text{mol product}\,{{i}}\text{ formed}}{\text{mol total soluble products formed}}{\cdot100}$$

$$\text{Selectivity of product }{{i}}\, {(\%)} \text{=} \frac{\text{mol electron-equivalents product}\,{{i}}\text{ formed}}{\text{mol electron-equivalents ethanol consumed}}{\cdot100}$$

## Results

First, we studied ethanol utilization by monocultures of *A. neopropionicum*, and odd-chain elongation by *C. kluyveri*. Next, a co-culture of *A. neopropionicum* and *C. kluyveri* was established in serum bottles, with ethanol and CO_2_ as sole substrates. The performance of the co-culture was further tested in a continuous bioreactor at increasing ethanol loading rates (ELRs).

### Effect of ethanol concentration on the growth and product profile of *A. neopropionicum*

**Fig. 1:**
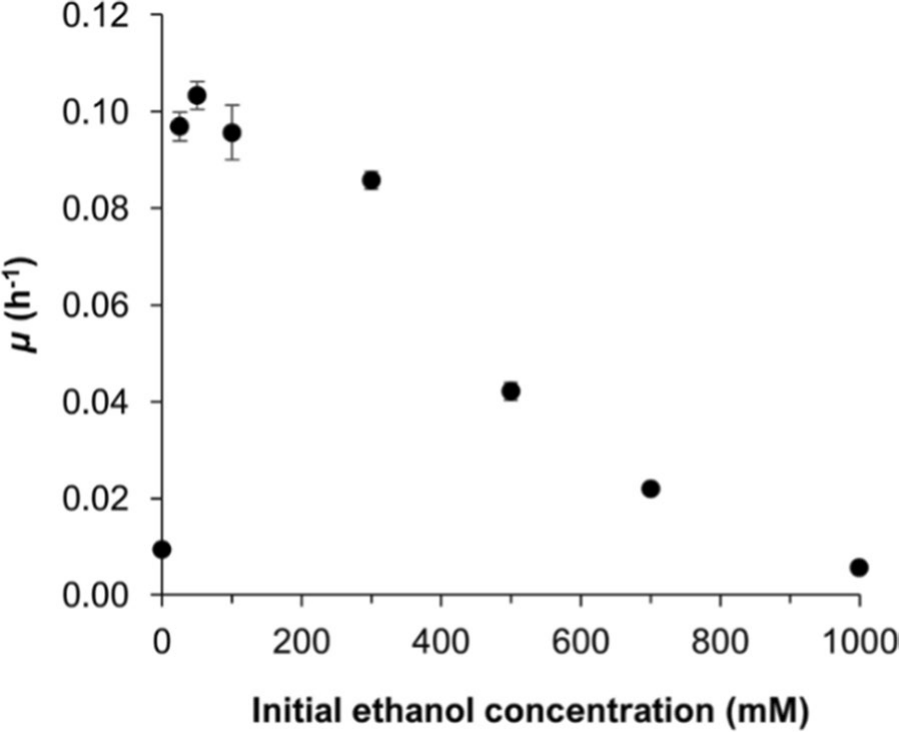
Effect of the ethanol concentration on the specific growth rate (*μ*) of *A. neopropionicum*. Growth rates were obtained from incubations with ethanol (0, 25, 50, 100, 300, 500, 700 mM and 1 M) and CO_2_ as substrates. Error bars indicate standard deviations of biological triplicates

Ethanol tolerance in *A. neopropionicum* was assessed by determining cell growth in serum bottles (batch growth) containing 25–1000 mM ethanol. Cell density and production profiles over time are presented in Additional file 1: Figs. S1, S2. Specific growth rates (*μ*) obtained under the different ethanol concentrations are shown in Fig. 1.

Growth of *A. neopropionicum* was most prominent with 50 mM ethanol, displaying a maximum specific growth rate (*μ*_max_) of 0.103 ± 0.003 h^−1^ (doubling time (*t*_*d*_) = 6.7 h) (Fig. 1). Slightly lower* μ* were obtained with ethanol concentrations of 25 mM (0.097 ± 0.003 h^−1^; *t*_*d*_ = 7.2 h) and 100 mM (0.096 ± 0.006 h^−1^; *t*_*d*_ = 7.2 h). Biomass formation was most prominent in this concentration range, with maximum cell densities of 59 ± 1 mg CDW L^−1^ (both with 50 mM and 100 mM ethanol) and 53 ± 1 mg CDW L^−1^ (with 25 mM ethanol). The *μ* declined to 0.086 ± 0.002 h^−1^ (*t*_*d*_ = 8.1 h) when the initial ethanol concentration was 300 mM; this titer is a threshold above which the growth rate of *A. neopropionicum* dropped sharply (Fig. 1). Cultivation in the absence of ethanol resulted in scarce biomass production (16 ± 2 mg CDW L^−1^), likely deriving from the utilisation of L-cysteine and yeast extract present in the medium. No significant growth was observed in bottles containing 1 M ethanol.

**Fig. 2:**
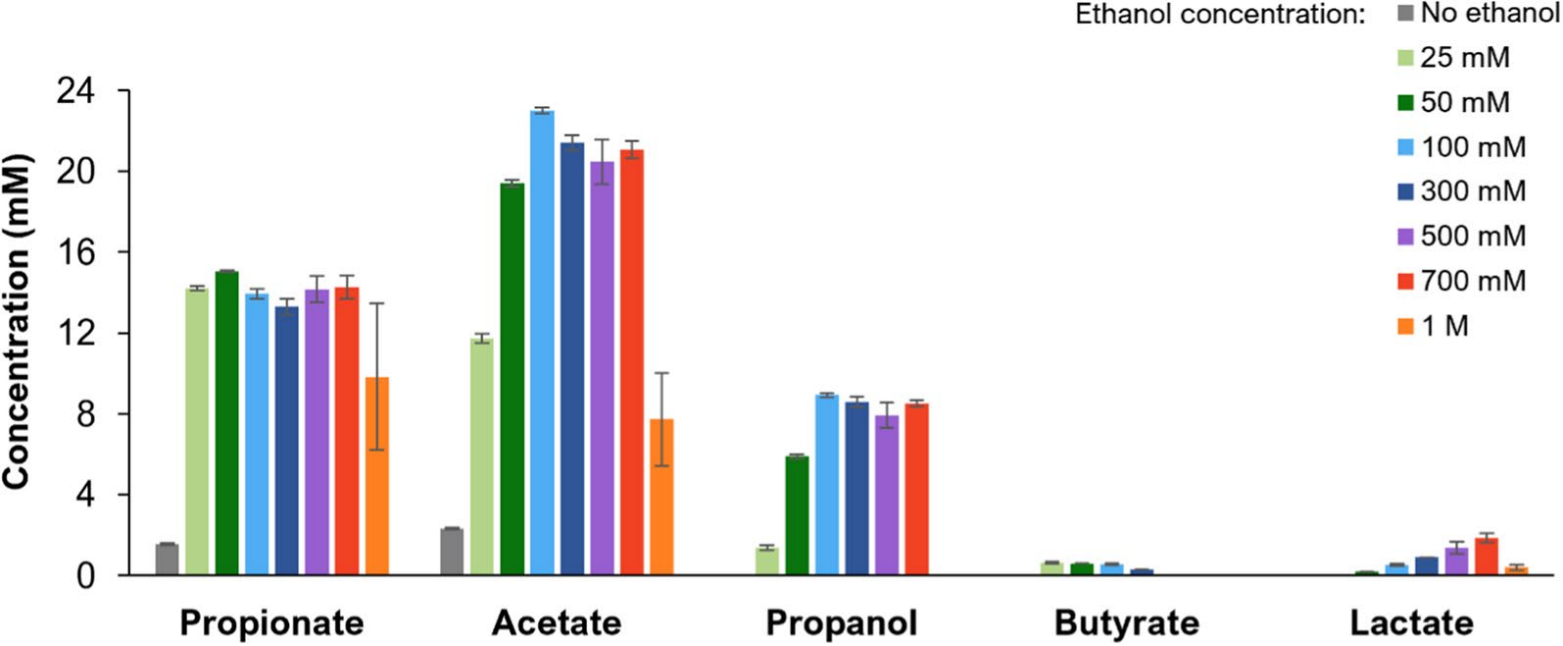
Product concentrations at the end of batch incubations of *A. neopropionicum* growing on ethanol (25–1000 mM) and CO_2_ as substrates. Error bars indicate standard deviations of biological triplicates

Product concentrations in the different incubations are shown in Fig. 2. Propionate and acetate were the two main products of ethanol fermentation by *A. neopropionicum*, with minor products being propanol, butyrate and lactate. An initial concentration of 25 mM ethanol yielded the highest propionate:acetate ratio, 1.2 (mol/mol). In this condition, cultures produced 14.2 ± 0.1 mM propionate and 11.7 ± 0.2 mM acetate. Incubations with 50 mM and 100 mM ethanol led to similar amounts of propionate (compared to 25 mM ethanol); however, acetate concentration doubled (to ≈ 21 mM). Higher initial ethanol concentrations (≥ 300 mM) yielded similar production of propionate and acetate, but also promoted the accumulation of secondary products, namely, propanol and lactate. Propanol specificity under 25 mM ethanol was 5% (1.4 ± 0.1 mM), yet it reached 19% (≈ 8.5 mM) in the incubations containing 100–700 mM ethanol. Formation of propanol occurred mostly during the stationary phase of growth and, with substrate concentrations > 50 mM ethanol, it coincided with a slight consumption of propionate (Additional file 1:Fig. S1). Lactate, on the other hand, was not detected with 25 mM initial ethanol, but it reached a specificity of 8% (1.9 mM ± 0.2 mM) in the bottles containing 700 mM ethanol. When produced, butyrate concentrations remained low (< 1 mM). It should be noted that ethanol was depleted only in the incubations with 25 mM ethanol (Additional file 1: Fig. S1). Incomplete conversion in assays with higher initial ethanol concentrations could be due to limitation of CO_2_/bicarbonate, acidification of the environment (final pH was 6.0–6.2, which is suboptimal for *A. neopropionicum* [54]) or substrate toxicity. Indeed, a substrate concentration of 1 M ethanol had a detrimental effect not only on growth, as depicted in Fig. 1, but also on the productivity of the cultures (Fig. 2).

To gain insight into the physiology of *A. neopropionicum*, we determined specific production/consumption rates (*q*) and product/biomass yields (*Y*) in the incubations containing 25–700 mM ethanol, which are summarised in Table 2. In all the incubations, propionate was formed at a faster rate than acetate and propionate yields were higher than acetate yields. The highest specific substrate uptake rate (*q*_S,max_) was obtained with 100 mM ethanol (64.9 ± 0.2 mmol ethanol g CDW^−1^ h^−1^). The specific production rates of propionate (*q*_*P*_) and acetate (*q*_*A*_) were the highest with 50 mM and 100 mM ethanol (no difference observed within the two conditions). Production rates were slightly lower in the incubations containing 25 and 300 mM ethanol for both propionate and acetate. An initial ethanol concentration of 500 mM or higher had a strong detrimental effect on both production rates.

**Table 2:** q-rates and yields in batch incubations of *A. neopropionicum* growing on ethanol

Ethanol (mM)	q-rates (mmol *i* g CDW^−1^ h^−1^)			Biomass yield (g CDW mol ethanol^−1^)	Product yield (mol *i* mol ethanol^−1^)		
	*q*_*S*_	*q*_*P*_	*q*_*A*_	*Y*_*X*_	*Y*_*P*_	*Y*_*A*_	*Y*_*PL*_
**25**	42.0 ± 0.6	21.1 ± 0.5	13.9 ± 0.2	1.91 ± 0.05	1.21 ± 0.03	0.38 ± 0.02	0.10 ± 0.01
**50**	53.0 ± 2.5	23.5 ± 0.8	14.4 ± 0.5	1.27 ± 0.03	0.78 ± 0.01	0.38 ± 0.00	0.40 ± 0.01
**100**	64.9 ± 0.2	23.7 ± 0.1	13.9 ± 0.1	0.93 ± 0.02	0.60 ± 0.01	0.34 ± 0.00	0.65 ± 0.01
**300**	ND	18.8 ± 0.8	9.6 ± 0.4	ND	ND	ND	ND
**500**	ND	10.4 ± 0.8	6.3 ± 0.6	ND	ND	ND	ND
**700**	ND	8.4 ± 0.5	4.5 ± 0.4	ND	ND	ND	ND

S: substrate (ethanol); P: propionate; A: acetate; X: biomass. PL: propanol

ND: not determined. Ethanol partly evaporated in incubations containing ≥ 300 mM ethanol; therefore, ethanol consumption could not be reliably quantified in those bottles, and the related parameters could not be calculated

Biomass and propionate yields (*Y*_*X*_ and *Y*_*P*_, respectively) were higher with lower initial ethanol concentrations. Thus, the highest *Y*_*X*_ (1.91 ± 0.05 g CDW mol ethanol^−1^) and *Y*_*P*_ (1.21 ± 0.03 mol propionate mol ethanol^−1^) were obtained in the bottles containing 25 mM ethanol. Interestingly, the acetate yield (*Y*_*A*_) was rather constant across the assays containing 25 to 100 mM ethanol (0.34–0.38 mol acetate mol ethanol^−1^). Higher ethanol concentrations led to higher yields of propanol and lactate; from 25 to 100 mM initial ethanol, the propanol yield (*Y*_*PL*_) increased from 0.1 to 0.7 (Table 2), and that of lactate from 0 to 0.96. In summary, increasing ethanol concentrations led to a reallocation of the substrate from biomass and propionate towards secondary products propanol and lactate.

### Comparison of propionate and acetate as electron acceptors during ethanol-driven chain elongation by *C. kluyveri*

Here, we studied the effect of the electron acceptor, acetate or propionate, on the cell growth and product spectrum of *C. kluyveri* during ethanol-based chain elongation. Five conditions were tested in serum bottles (batch). In three of them, the ethanol:carboxylic acid ratio (E/CA; mol/mol) was fixed at 1.2, which corresponds to the theoretical stoichiometry of chain elongation for optimal substrate use [24]. These tests were performed with ethanol (120 mM) and either acetate (100 mM), propionate (100 mM) or both SCCAs (50 mM each) present. To assess the effect of the SCCA concentration, two additional sets were established with ethanol (120 mM) and either acetate or propionate at 50 mM (E/CA = 2.4). Table 3 summarises the growth-related parameters obtained in the five conditions tested. Production profiles of all incubations can be found in Additional file 1: Fig. S3.

Ethanol was depleted in all incubations except for in the bottles with 50 mM propionate, where ∼ 13 mM ethanol remained (Additional file 1: Fig. S3). The presence of one or another SCCA had a strong impact on the lag phase of the cultures. In all the incubations with acetate, the lag phase was ≤ 17 h [17 h corresponds to first sampling point after t_0_, and at this point cells were already growing exponentially (Additional file 1: Fig. S4)]. In contrast, when cultures were initiated with only propionate (50 mM and 100 mM), the lag phases were 60 h and 66 h, respectively. The presence of acetate or propionate also influenced the growth rate of *C. kluyveri*. Incubations containing 100 mM and 50 mM acetate showed the fastest growth with a *μ* of 0.122 ± 0.003 h^−1^ (*t*_*d*_ = 5.7 h) and 0.112 ± 0.005 (*t*_*d*_ = 6.2 h), respectively. Growth rates decreased approximately 1.8 times with propionate as sole electron acceptor, with no difference observed within the two propionate concentrations tested (Table 3). However, when acetate was supplied next to propionate in equimolar amounts, the growth rate was similar to the cultures containing only acetate. Biomass formation also differed depending on the electron acceptor supplied. Incubations supplied with acetate as sole electron acceptor produced approximately three times more biomass per mol of ethanol consumed than those with only propionate (Table 3). In both cases, a higher E/CA ratio of 2.4 resulted in a higher Y_X_ (1.4-fold increment for both acetate and propionate incubations, compared to an E/CA of 1.2). With acetate only, maximum cell densities were 62 ± 5 mg CDW L^−1^ (100 mM acetate, E/CA = 1.2) and 90 ± 7 mg CDW L^−1^ (50 mM acetate, E/CA = 2.4). Significantly lower cell densities were obtained with only propionate, i.e., 26 ± 3 mg CDW L^−1^ (100 mM propionate, E/CA = 1.2) and 35 ± 4 mg CDW L^−1^ (50 mM propionate, E/CA = 2.4). However, similar to what was observed with the growth rates, when both electron acceptors were present (50 mM each, E/CA = 1.2), biomass production (79 ± 10 mg CDW L^−1^) was identical to tests with acetate only.

**Table 3:** Growth-related parameters of *C. kluyveri* batch growth with ethanol (120 mM) plus the indicated SCCA

Electron acceptor (E/CA)	Lag phase (*h*)	μ (h^−1^)	*Y*_*X*_ (g CDW mol ethanol^−1^)
Acetate 100 Mm (1.2)	17^a^	0.122 ± 0.003	0.59 ± 0.03
Acetate 50 mM (2.4)	17^a^	0.112 ± 0.005	0.83 ± 0.09
Acetate 50 mM + Propionate 50 mM (1.2)	17^a^	0.109 ± 0.007	0.75 ± 0.10
Propionate 100 mM (1.2)	66	0.071 ± 0.002	0.24 ± 0.03
Propionate 50 mM (2.4)	60	0.065 ± 0.006	0.33 ± 0.06

*E/CA* ethanol:carboxylic acid ratio (mol/mol). ^a^The first sample was taken after 17 h, when cells were already exponentially growing (Additional file 1: Fig. S4). Therefore, the lag phase in these cultures was likely to be < 17 h

**Fig. 3:**
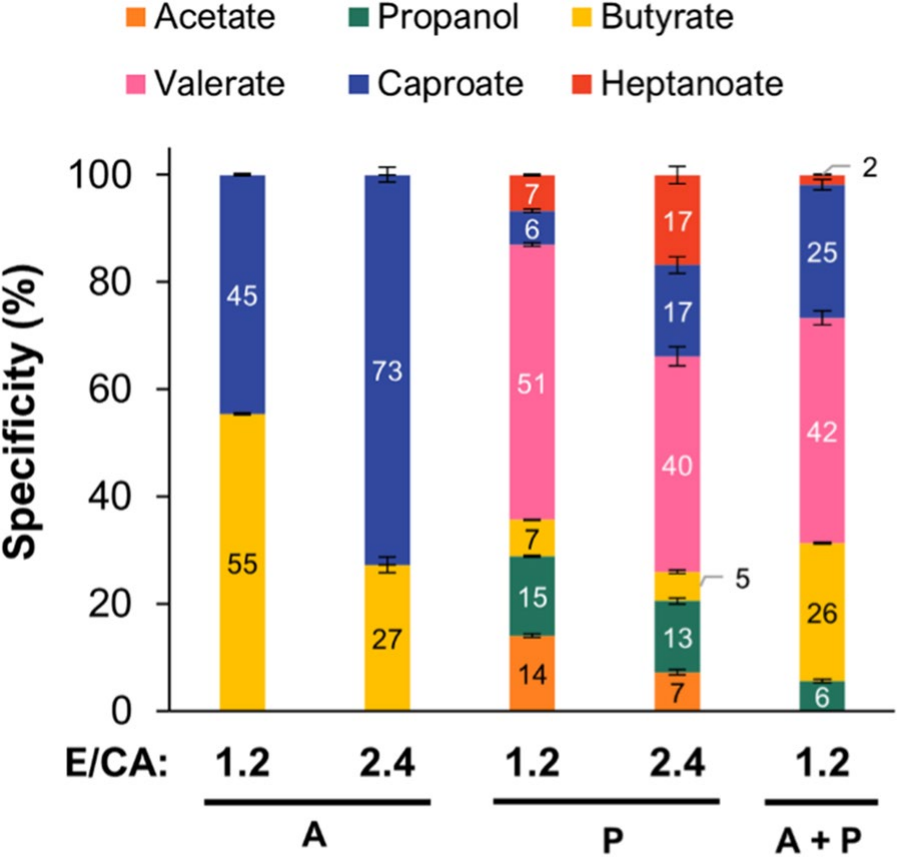
Product specificities at the end of batch incubations of *C. kluyveri* grown on ethanol (120 mM) plus the indicated SCCA(s). *E/CA* ethanol:carboxylic acid ratio (mol/mol). A: acetate; P: propionate. Error bars indicate standard deviations of biological triplicates

Product specificities (%) at the end of batch cultivations are depicted in Fig. 3. As expected, *C. kluyveri* produced only even-chain MCCAs when acetate was the only electron acceptor. With an E/CA ratio of 1.2 (100 mM acetate), butyrate specificity was 55% (33.8 ± 1.5 mM) and that of caproate, 45% (27.3 ± 1 mM). Caproate became more abundant with an E/CA ratio of 2.4 (50 mM acetate), reaching a specificity of 73% (40.6 ± 4.8 mM). Incubations with propionate resulted in the production of valerate as dominant product. With an E/CA of 1.2 (100 mM propionate), valerate specificity was 51% (54.9 ± 1 mM), and heptanoate represented 7% of the products (7.4 ± 0.4 mM). Even-chain carboxylic acids (acetate, butyrate and caproate) were also produced; in total, these accounted for 27% of the products. Propanol was also present (15%, 15.8 ± 0.4 mM), contributing to an overall odd-chain specificity of 73% (OCCAs: 58%). Similar to what was observed in acetate incubations, increasing the E/CA ratio to 2.4 (50 mM propionate) favoured the production of the longer chain carboxylates, specifically heptanoate (17%, 14 ± 1 mM) and caproate (17%, 13.9 ± 1.2 mM) at the expense of their respective precursors (valerate and butyrate). Yet, valerate was the dominant carboxylic acid (40%, 33.2 ± 2.6 mM). Overall, the specificity of odd-chain products was 70% (OCCAs: 57%).

When both propionate and acetate were supplied (50 mM each, E/CA = 1.2), *C. kluyveri* produced a mixture of odd- and even-chain products. Propionate and acetate were used simultaneously (Additional file 1: Fig. S3c). The most abundant product was valerate, with a specificity of 42% (30.4 ± 0.7 mM); however, butyrate and caproate together accounted for a higher proportion (37.3 mM in total, 51%). This condition yielded the lowest amount of heptanoate (1.3 ± 0.1 mM, 2%). There was no difference in H_2_ production (~ 50 kPa) across all the conditions tested.

### Synthetic co-culture of ***A. neopropionicum*** and ***C. kluyveri*** producing odd- and even-chain MCCAs from ethanol and CO_2_

**Fig. 4:**
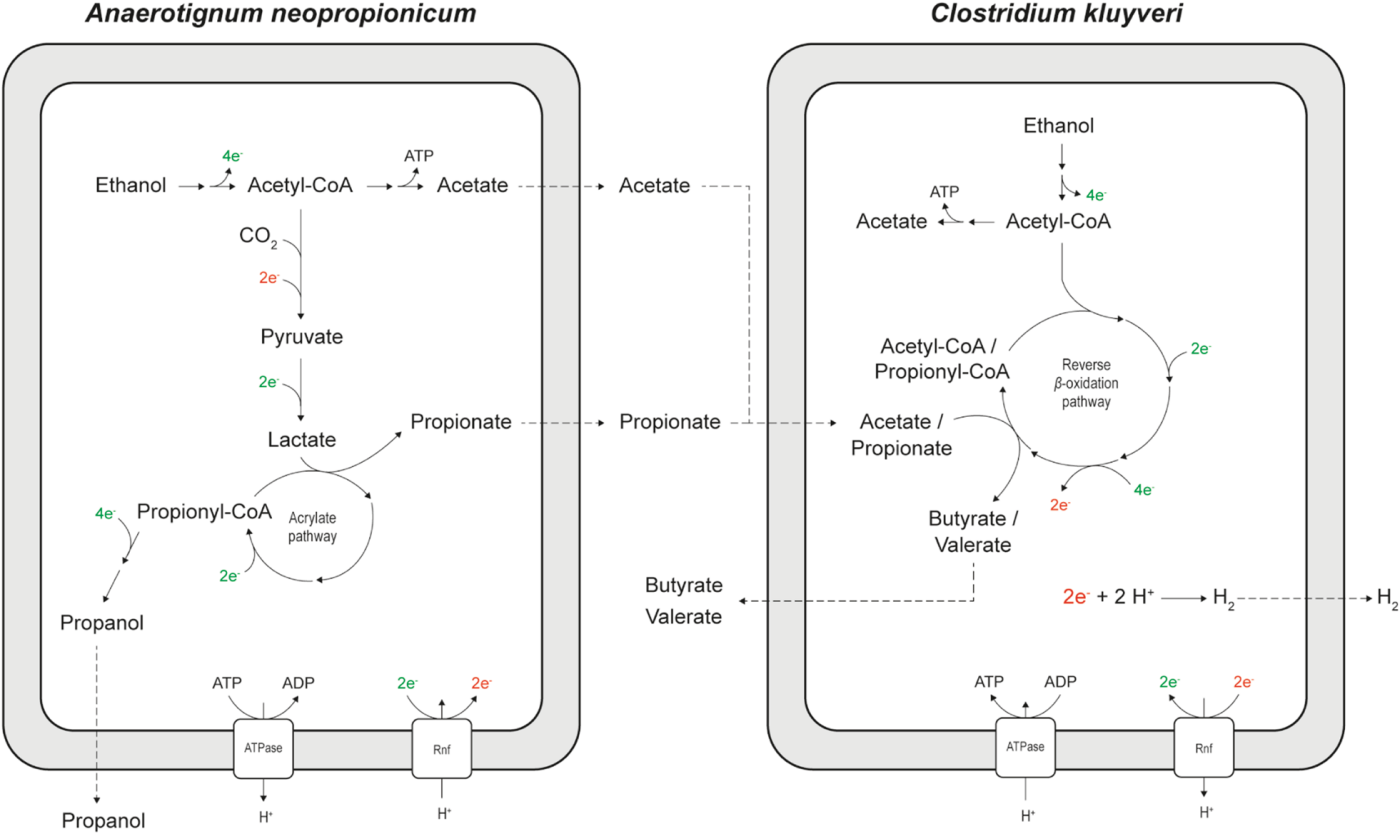
Schematic representation of the *A. neopropionicum*–*C. kluyveri* co-culture. Stoichiometry of the reactions and ATP yield are not shown. Dashed lines indicate metabolite transport or diffusion across the cell. Reducing equivalents in green and in red indicate NADH/NAD(P)H and reduced ferredoxin, respectively. Rnf: ferredoxin:NAD^+^ oxidoreductase complex

The synthetic co-culture of *A. neopropionicum* and *C. kluyveri* was established with ethanol and CO_2_ as sole substrates. A scheme of the co-culture depicting the main conversions and metabolites involved is displayed in Fig. 4.

**Fig. 5:**
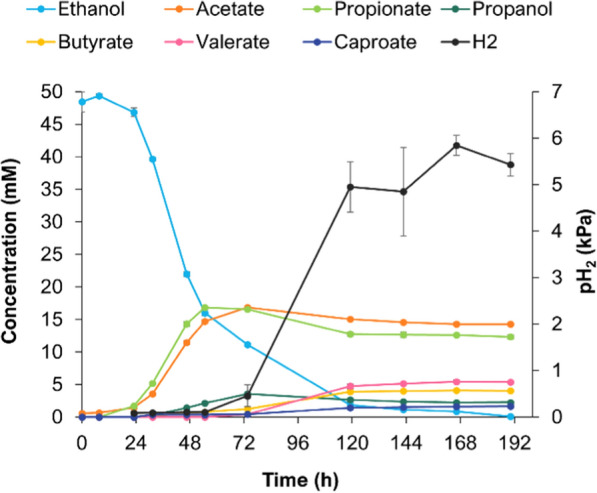
Ethanol and product profiles of the *A. neopropionicum*–*C. kluyveri* co-culture in serum bottles, with ethanol (50 mM) and CO_2_ (not shown) as substrates. Error bars indicate standard deviations of biological triplicates

The co-culture was initiated with 50 mM ethanol. Figure 5 shows substrate consumption and production profile over time. At day 5 of incubation (120 h), ethanol was depleted and the co-culture had produced 5.4 ± 0.1 mM valerate, 4.0 ± 0.1 mM butyrate, 1.6 ± 0.1 mM caproate and trace amounts of heptanoate (< 0.5 mM). Ethanol was used by both species, although not simultaneously; two phases could be distinguished in ethanol consumption that are linked to growth of the two microorganisms, as described hereafter. In the first phase, lasting about 55 h, most of the ethanol was consumed (~ 32 mM), and propionate and acetate were produced simultaneously. The almost equimolar amounts of propionate and acetate formed fit with the stoichiometry observed in pure cultures of *A. neopropionicum* (Additional file 1: Fig. S1b). During this phase no H_2_ was produced, an indication that *C. kluyveri* was not yet active. Low amounts of propanol and butyrate (≤ 2 mM) were detected, which could have been produced by *A. neopropionicum* as this was also observed in pure culture tests (Fig. 2). In the second phase (55 h onwards), the remaining ethanol (~ 17 mM) was used for chain elongation by *C. kluyveri*. Acetate and propionate were consumed simultaneously, following the same pattern observed in pure culture incubations of *C. kluyveri* (Additional file 1: Fig. S3c). The apparent lower consumption of acetate compared to propionate can be explained by endogenous acetogenesis by this strain (1/6th of ethanol, according to theoretical stoichiometry [24]). Due to limited availability of ethanol in this second phase, only a fraction of the SCCAs was consumed, thus ~ 12 mM propionate and ~ 14 mM acetate remained unused. In this period, H_2_ was produced concomitantly with the chain-elongated products to a final partial pressure of 5.5 kPa. Clearly, growth of *A. neopropionicum* was dominant in the co-culture; most of the ethanol was consumed in the first 55 h of incubation by this species (Fig. 5). Despite this resulting in acetate and propionate as main final products of the co-culture, and not MCCAs, this experiment demonstrated the feasibility of C4–C7 carboxylates production by the *A. neopropionicum*–*C. kluyveri* co-culture from solely ethanol and CO_2_ as substrates. Incubation in batch presented two main limitations: i) limited availability of ethanol for chain elongation due to faster consumption by *A. neopropionicum,* and ii) limited buffering capacity (pH at the end of cultivations was 5.9), which could have played an unfavourable role on the growth of both strains. Thus, the co-culture was further on studied in a pH-controlled bioreactor system with continuous ethanol supply.

### Continuous production of C4–C7 carboxylates from ethanol and CO_2_ by the ***A. neopropionicum***–***C. kluyveri*** co-culture in a chemostat bioreactor

**Fig. 6:**
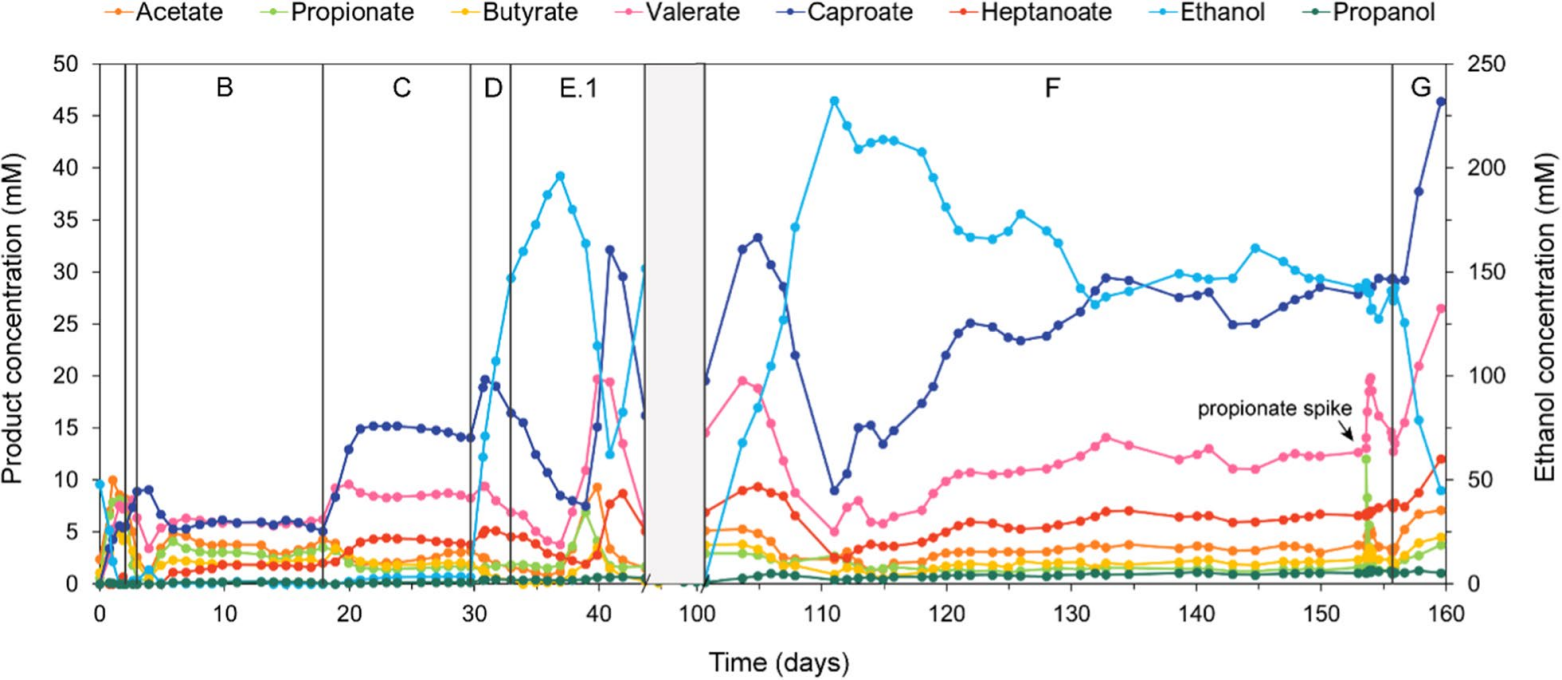
Product concentrations (left axis) during continuous fermentation of ethanol (right axis) by the *A. neopropionicum*–*C. kluyveri* co-culture. The relevant phases B–E.1, F and G are indicated. The shaded area corresponds to a phase (E.2) where several technical issues occurred and is, therefore, disregarded. The arrow points to the moment we spiked the system with 10 mM propionate

The productivity of the *A. neopropionicum*–*C. kluyveri* co-culture was tested in a CSTR under a continuous flow of N_2_/CO_2_ and at increasing ethanol loading rates (ELRs). The bioreactor was operated at 35 °C and the pH maintained at 7. Ethanol concentration in the inflow varied from 50 to 300 mM (Table 1) to simulate the range of ethanol concentrations in syngas fermentation effluent [55, 56]. Microscopic inspection of the culture at different timepoints during the run confirmed the presence of the two strains and the absence of contamination. The bioreactor was started in batch for about 2 days, after which continuous operation was initiated with an ELR of 1.7 g ethanol L^−1^ d^−1^ and 50 mM ethanol in the inflow (phase A). On day 3, the gas flowrate was increased to 3 L h^−1^ (0.07 vvm; phase B). A steady state was reached during this phase, with the production of C4–C7 carboxylates (Fig. 6). This demonstrated that chain elongation activity by *C. kluyveri* was sustained by cross-feeding of SCCAs (i.e., acetate and propionate) produced by *A. neopropionicum*. Ethanol consumption during steady-state B was high (99.3%), indicative that the system was ethanol-limited. Production of C4–C7 carboxylates in this steady-state and subsequent ones is summarised in Table 4. Valerate and caproate were equally abundant in phase B, each representing a quarter of the products and with steady-state concentrations of 5.9 ± 0.2 mM and 5.8 ± 0.3 mM, respectively. Their observed production rates were also similar (~ 4 mmol L^−1^ d^−1^). Heptanoate was also produced, yet at the lowest rate of all the carboxylic acids (1.2 ± 0.1 mmol L^−1^ d^−1^).

**Table 4:** Averaged production values of C4–C7 carboxylates in the three steady states (in phases B, C and F) of the bioreactor cultivation

Steady-state parameter	Phase	ELR (g L^−1^ d^−1^)	HRT (h)	Carboxylic acids			
				Butyrate	Valerate	Caproate	Heptanoate
Concentration (mM)	B	1.7	36	2.1 ± 0.2	5.9 ± 0.2	5.8 ± 0.3	1.7 ± 0.2
	C	3.1	36	1.9 ± 0.1	8.5 ± 0.2	14.8 ± 0.5	4.1 ± 0.2
	F	6.7	54	2.1 ± 0.2	12.5 ± 0.8	27.6 ± 1.3	6.5 ± 0.3
Observed prod. rate (mmol L^−1^ d^−1^)	B	1.7	36	1.4 ± 0.1	4.0 ± 0.1	3.9 ± 0.2	1.2 ± 0.1
	C	3.1	36	1.3 ± 0.1	5.7 ± 0.1	9.9 ± 0.3	2.8 ± 0.1
	F	6.7	54	0.9 ± 0.1	5.5 ± 0.4	12.2 ± 0.6	2.9 ± 0.1
% Specificity (mol_i_/mol_total products_)	B	1.7	36	9.2 ± 1.1	26.3 ± 0.9	25.8 ± 1.9	7.6 ± 0.8
	C	3.1	36	5.6 ± 0.3	24.8 ± 0.2	43.3 ± 1.4	12.2 ± 0.7
	F	6.7	54	3.4 ± 0.4	20.9 ± 0.6	46.3 ± 0.5	10.9 ± 0.2
% Selectivity (e-mol_i_/e-mol_eth. consumed_)	B	1.7	36	6.7 ± 0.5	25.2 ± 0.7	30.4 ± 1.8	10.7 ± 1.2
	C	3.1	36	3.5 ± 0.2	19.9 ± 0.4	42.7 ± 1.2	14.2 ± 0.8
	F	6.7	54	2.0 ± 0.2	15.7 ± 0.7	42.8 ± 1.5	11.9 ± 0.4

*ELR* ethanol loading rate

As indicated by the product selectivity, most electrons from the substrate (ethanol) ended up in caproate (30.4 ± 1.8%) and valerate (25.2 ± 0.7%). Overall, an equal proportion of odd- and even-chain MCCAs were produced during steady-state B, as indicated by their respective specificities (34.0 ± 1.2% and 34.9 ± 2.6%).

To boost chain elongation activity, the concentration of ethanol in the inflow was doubled (95 mM), resulting in an ELR of 3.1 g ethanol L^−1^ d^−1^ (phase C). A new steady-state was reached (Fig. 6), in which ethanol consumption remained high (96.6%) and the biomass density increased from 75 ± 13 mg CDW L^−1^ (O_600_ ~ 0.4) to 106 ± 15 mg CDW L^−1^ (OD_600_ ~ 0.6) (Additional file 1: Fig. S5). Compared to phase B, steady-state concentrations of caproate and heptanoate more than doubled, and caproate productivity (9.9 ± 0.3 mmol L^−1^ d^−1^) surpassed valerate productivity (5.7 ± 0.1 mmol L^−1^ d^−1^) (Table 4). The concentration of valerate also increased, while that of butyrate was practically unchanged. The selectivity of the C6 (42.7 ± 1.2%) and C7 (14.2 ± 0.8%) carboxylates increased with respect to phase B, indicating a higher allocation of electrons from ethanol being used for chain elongation. The most abundant product during ss–C, based on specificity, was caproate (43.3 ± 1.4%), followed by valerate (24.8 ± 0.2%) and heptanoate (12.2 ± 0.7%). Compared to the previous phase, the specificity of even-chain products (C4 + C6) increased to 48.9 ± 1.3%, a 29% increment, while that of OCCAs (C5 + C7) rose to 37.0 ± 0.7%, a much lower 8% increment.

To test the robustness of the co-culture, on day 30, the ELR was set to 8.0 g ethanol L^−1^ d^−1^ by increasing the ethanol concentration in the inflow to 300 mM and the HRT to 42 h (phase D). Cell density declined sharply in the following days (Additional file 1: Fig. S5) and ethanol accumulated up to 147 mM (Fig. 6). In an attempt to recover the system, on day 34 the HRT was extended to 54 h, resulting in an ELR of 6.3 g ethanol L^−1^ d^−1^ (phase E.1). A few days later, the culture recovered its chain-elongating activity, as denoted by an increase of the concentration of C4–C7 carboxylic acids, which peaked around day 40 (caproate: 32.1 mM; valerate: 19.7 mM; heptanoate: 8.7 mM). Cell density also peaked, at an OD_600_ of 1.2 (Additional file 1: Fig. S5). However, perhaps due to the fast accumulation of MCCAs, the co-culture performance declined around day 41: at this point, ethanol accumulated and cell density declined sharply, reaching an OD_600_ of ~ 0.5 on day 44. Unfortunately, technical issues occurred in the following weeks (phase E.2): first, on day 55 the outflow line clogged. Second, ethanol in the medium tank (that was placed at room temperature and continuously flushed with N_2_) was slowly being stripped out. The latter technical fault went undetected for several weeks. These issues altered the culture behaviour and made it not possible to rely on the obtained data. For this reason, phase E.2 is disregarded (shaded area in Fig. 6 and in Additional file 1: Fig. S5). Subsequently, a condenser was connected to the medium tank, which prevented further ethanol loss via gas stripping.

The system was properly functioning again on day 100 at an ELR of 6.7 g ethanol L^−1^ d^−1^ (phase F), with an ethanol concentration in the inflow of 319 mM. Likely, the higher ethanol loading rate and inflow concentration slowed down growth of both strains - particularly *A. neopropionicum* as seen in pure culture (Fig. 1) -, resulting in a rather long acclimation time (≈ 30 days) before the culture stabilised. Initially, ethanol accumulated, reaching 232 mM, accompanied by a biomass density drop (from OD_600_ 0.8 to 0.3) and a decline in MCCAs concentrations (Fig. 6). However, chain elongation activity recovered on day 111 and, around day 131, the system reached the third steady state, in phase F. Ethanol consumption during this period was 54.1%, about half compared to phases B and C. Nevertheless, MCCA production increased, with the concentrations of valerate (12.5 ± 0.8 mM, 21%), caproate (27.6 ± 1.3 mM, 47%) and heptanoate (6.5 ± 0.3 mM, 11%) being the highest of the three steady states. Propanol, which can be produced by both strains, became significantly more abundant in this phase, reaching a specificity of 8.4 ± 0.5% (5.0 ± 0.4 mM), in contrast to ≈ 2% in phases B and C. Lactate was also detected in this phase (~ 0.8 mM). The specificity of OCCAs (C5 + C7) in phase F, 31.8 ± 0.7%, was the lowest of the three periods, while that of even-chain products (C4 + C6), 49.7 ± 0.5%, was the highest.

Acetate and propionate concentrations were rather low in phase F (< 4 mM). To test whether shortage of intermediates was limiting chain elongation activity, on day 153 we spiked the system with 10 mM propionate. In the hours following the spike, valerate concentration quickly rose to 19.8 mM before returning to the pre-spike level (Fig. 6). This observation led us to increase the pH set point from 7 to 7.3 (phase G), optimum for *A. neopropionicum* [54] and also favourable to decrease toxicity by accumulation of carboxylic acids. This change immediately triggered ethanol consumption and a significant increase of the concentration of all carboxylic acids. The bioreactor system had to be shut down shortly after due to logistical reasons, just before the highest concentrations of caproate (46.4 mM), valerate (26.5 mM) and heptanoate (12.0 mM) were recorded.

## Discussion

In this work, we showed that a mixture of C5–C7 MCCAs can be produced from solely ethanol and CO_2_ using a microbial co-cultivation approach. The designed system combines the propionigenic bacterium *A. neopropionicum*, which grows on ethanol producing propionate and acetate, and the chain-elongating microorganism *C. kluyveri* that uses the produced short-chain carboxylates as acceptor molecules for chain elongation (Fig. 4). Despite the fact that both strains use ethanol as substrate, during continuous cultivation they established an interaction based on cross-feeding of SCCAs, which allowed the co-culture to sustain over time (Fig. 6). We envision that this co-culture could be applied to upgrade dilute ethanol from syngas fermentation effluent to odd-numbered carbon MCCAs.

### Effect of ethanol concentration on the co-culture viability

We first investigated ethanol tolerance of *A. neopropionicum.* Syngas fermentation systems yield ethanol titres ranging 0.1–0.4 M (5–20 g L^−1^) [55, 56], with the highest reported concentration being 1 M (48 g L^−1^) [57]. Microbial strains used for upgrading of syngas fermentation effluent should tolerate and use ethanol in this range of concentrations. Ethanol is known for its adverse effects to microorganisms [58]. While ethanol tolerance of *C. kluyveri* has been extensively investigated [21, 23, 39, 59, 60] and demonstrated to support robust growth at concentrations as high as 700 mM ethanol [59, 60], the impact of ethanol toxicity on *A. neopropionicum* or other propionibacteria remained unexplored. Our experiments showed that *A. neopropionicum* grows optimally with 25–100 mM ethanol (1.2–4.6 g L^−1^) and can tolerate well ethanol concentrations up to 300 mM (13.8 g L^−1^) (Fig. 1). These findings are significant as these ethanol concentrations are within the range typically present in effluents from syngas fermentation. The *A. neopropionicum*–*C. kluyveri* co-culture was functional under substrate loading rates of 1.7–6.7 g ethanol L^−1^ d^−1^ and inflow concentrations of up to 300 mM ethanol. While higher ethanol concentrations could have been tolerated by *C. kluyveri*, we did not test those as they would have likely been detrimental to the co-culture due to the inhibitory effect on *A. neopropionicum*.

### Continuous production of C5–C7 carboxylates from ethanol and CO_2_ by the synthetic co-culture

Most studies so far addressing the conversion of dilute ethanol to MCCAs have targeted the production of even-chain products, either by open mixed cultures or by pure cultures of *C. kluyveri*. Here, we chose an alternative approach, a synthetic co-culture, to target the production of OCCAs. The rationale for this approach was to: i) avoid unwanted reactions, e.g., methanogenesis, which normally take place when using open mixed cultures, ii) incorporate a bacterium able to produce propionate, the key precursor of OCCAs, and iii) cultivate together strains that grow optimally at similar environmental conditions (i.e., temperature, pH).

The *A. neopropionicum*–*C. kluyveri* co-culture produced a maximum of 37.0 ± 0.7% OCCAs (C5 + C7), achieved with an ELR of 3.1 g ethanol L^−1^ d^−1^ (phase C). Under these conditions, valerate accounted for a significant 25% of the total products. The maximum steady-state concentrations of valerate (12.5 ± 0.8 mM or 1.3 g L^−1^) and heptanoate (6.5 ± 0.3 mM or 0.85 g L^−1^) were obtained under the highest substrate loading rate (6.7 g ethanol L^−1^ d^−1^; phase F). While these titres were higher than in the previous period where a lower ELR was applied (3.1 g ethanol L^−1^ d^−1^; phase C), the production rates of the two compounds remained the same. This suggests that the higher valerate and heptanoate concentrations observed in phase F could be due to the longer HRT imposed (54 h, vs. 36 h in phase C). On the contrary, caproate production increased with higher ELRs (Table 4). Conversely to phases B and C, ethanol utilisation was not complete (54.1%) in phase F. Overall, this seems to indicate that such scenario where ethanol was not limiting could have driven excessive ethanol oxidation to acetate by *C. kluyveri*, leading to predominance of even-chain elongation activity. Indeed, caproate was the dominant product in phase F (46.3 ± 0.5%) and it was produced at the fastest rate (12.2 ± 0.6 mmol L^−1^ d^−1^). The specificity of OCCAs decreased in phase F (31.8 ± 0.7%) with respect to the previous period (phase B) and was actually the lowest within the three steady states (Table 4).

The selectivity of valerate, heptanoate and butyrate also dropped in phase F (Table 4), while a substantial fraction of electrons from the substrate was directed to propanol. Propanol production can be attributed to either *A. neopropionicum* [53, 54] and/or *C. kluyveri* [23, 51]. Propanol can be formed via reduction of propionyl-CoA [53, 61] or via reduction of propionate, as our batch experiments hinted (Fig. 5 and Additional file 1: Fig. S1). The monoculture experiments with *A. neopropionicum* showed that the yield of propanol increased when higher initial ethanol concentrations were used (Table 2). This observation was concurrent with high acetate and propionate titres, which accounted for approximately 30 mM in those conditions (Fig. 2). In the reactor, the concentration of total carboxylic acids in steady-state F was approximately 54 mM (Table 4). Thus, the increased production of propanol by *A. neopropionicum* could have been a mechanism to mitigate undissociated acid toxicity by decreasing the concentration of propionate and preventing further acidification of the environment. Previously, de Leeuw et al. [43] speculated that *C. kluyveri* reduced carboxylates to alcohols as a way to dispose of excess reducing equivalents under acetate limitation. However, in our reactor, the acetate concentration remained relatively constant at approximately 3 mM during the three steady states (phases B, C and F), whereas propanol levels increased significantly only during the latter period (Fig. 6). Therefore, it appears more plausible that any potential involvement of *C. kluyveri* in propanol formation would have been driven by the accumulation of carboxylic acids in the fermentation broth. Both strains are also able to consume propanol [23, 54, 62], so it is plausible that the actual propanol formation in the co-culture was higher than we measured. *A. neopropionicum* grows well on propanol in the presence of acetate and CO_2_, yielding propionate as end product [54, 62]. On the other hand, *C. kluyveri* can use propanol for chain elongation analogously to ethanol [23]. However, we believe that propanol-driven chain elongation may have contributed very little to MCCA production in our co-culture, as *C. kluyveri* has been reported to favour the utilization of ethanol over propanol when both substrates are present [23]. Besides propanol, lactate was detected under the highest ELR tested (6.7 g ethanol L^−1^ d^−1^). Lactate is an intermediate of the acrylate pathway that *A. neopropionicum* uses to metabolise ethanol [53, 54]; therefore, its accumulation points to a metabolic bottleneck at high substrate concentrations, as observed in pure culture incubations (Fig. 2).

Continuous OCCAs production through ethanol-based chain elongation has also been studied by Grootscholten et al. [49], who achieved an OCCAs selectivity of 57%, and by Roghair et al. [50], who reported similar values. Both studies relied on the use of mixed cultures. The highest valerate concentration (12.5 ± 0.8 mM or 1.3 g L^−1^) and productivity (5.7 ± 0.1 mmol L^−1^ d^−1^ or 0.58 g L^−1^ d^−1^) achieved by the *A. neopropionicum*–*C. kluyveri* co-culture in our study are lower than those achieved by Grootscholten et al., who reported a valerate concentration of 51.9 mM (5.3 g L^−1^) with at a production rate of 73.3 mmol L^−1^ d^−1^ (7.5 g L^−1^ d^−1^) [49]. The authors fed 13 g ethanol L^−1^ d^−1^ into their reactor, a much higher loading rate than applied in our system (1.7–6.7 g ethanol L^−1^ d^−1^). They also continuously supplemented the culture with propionate (10.4 g L^−1^ d^−1^), which we did not do here as we relied solely on propionate formed by *A. neopropionicum*. By increasing the loading rate to 19.5 g ethanol L^−1^ d^−1^, Grootscholten et al. also achieved the highest heptanoate productivity in an ethanol-driven chain elongating system, with a titre of 24.6 mM (3.2 g L^−1^) at a rate of 34.6 mmol L^−1^ d^−1^ (4.5 g L^−1^ d^−1^) [49]. Roghair et al. also supplied high substrate loading rates (32.5 g ethanol L^−1^ and 10.9 g propionate L^−1^ d^−1^, respectively) that led to the formation of 9.2 mM heptanoate (1.2 g L^−1^) with a productivity of 13.8 mmol L^−1^ d^−1^ (1.8 g L^−1^ d^−1^) [50].

Although the production rates of OCCAs obtained in our study are an order of magnitude lower than the benchmark achieved by Grootscholten et al. [49], they are superior than reported for a mixed culture in one-pot production from CO [29]. In that study, He and co-authors demonstrated the potential of integrating syngas fermentation and chain elongation in a single reactor. However, the titres of OCCAs they obtained were below 2 mM, with maximum valerate and heptanoate production rates of 0.83 mmol L^−1^ d^−1^ and 0.44 mmol L^−1^ d^−1^, respectively [29]. In addition, a long acclimation time (∼100 days) preceded the production of MCCAs, as observed in similar mixed culture processes [18, 29, 39, 63]. In contrast, the *A. neopropionicum*–*C. kluyveri* co-culture presented in this work reached steady production of C5–C7 carboxylates in just a few days when ethanol was fed at 1.7 and 3.1 g L^−1^d^−1^ (Fig. 6). This suggests that, while integration of syngas fermentation and chain elongation in a single step has its advantages (e.g., operation of one bioreactor instead of two), separating the two processes might allow for higher production rates of carboxylic acids.

### Insights into the metabolism of odd-chain elongation by *C. kluyveri* and excessive ethanol oxidation

Early studies on *C. kluyveri* showed that propionate is as good electron acceptor as acetate for chain elongation [21, 23]. The effect of the ethanol:propionate ratio on the product spectrum has been recently studied [51]. Our monoculture experiments with *C. kluyveri* aimed to contribute to the knowledge on odd-chain elongation in this microorganism, focusing on cell growth. The longer lag phase and lower growth rate observed with propionate, compared to growth on acetate (Table 3), could be explained by several reasons. On one hand, it could be due to a higher toxicity of propionate or, more likely, propionyl-CoA, compared to acetate/acetyl-CoA. In *C. kluyveri*, propionate (as well as acetate) is metabolised by the enzyme butyryl-CoA:acetate CoA transferase (Cat3), yielding a propionyl-CoA molecule that is fed into the reverse β-oxidation cycle [24]. Propionyl-CoA is toxic when accumulated inside the cell, retarding growth and inhibiting biosynthetic reactions through inhibition of CoA-dependent enzymes [64–67]. Bornstein and Barker [21] reported some inhibition of *C. kluyveri* grown with propionate concentrations above 68 mM, which are in the range of the levels tested in our study (50 and 100 mM). It could also be hypothesised that the slower growth observed with propionate is due to a lower affinity of the enzymes involved in the reverse β-oxidation pathway for odd-numbered intermediates, compared to even-numbered intermediates. However, in our experiments, acetate and propionate were used simultaneously when they were both initially present (Additional file 1: Fig. S3c), suggesting no substrate preference. This is in line with observations by Candry et al. [51] in their study of odd-chain elongation in *C. kluyveri*. The authors of that study also reported no difference in the specific growth rate of *C. kluyveri* with propionate or acetate as electron acceptors, which contradicts our results (Table 3). This discrepancy could be due to the use of different media or, perhaps, the fact that the ethanol concentration (343 mM) and ethanol:carboxylic acid ratio (3.4) in the study of Candry et al. [51] were much higher than in our work; excess ethanol can be oxidised to acetate with concomitant ATP production, supporting growth. In our study, we also observed that the adverse effects of propionate on cell growth and biomass formation were overcome when acetate was present (Table 3). This hints at a shortage of acetyl-CoA, key metabolic intermediate derived from acetate and ethanol, as the cause for the lower growth rates and biomass yields observed with propionate. In other words, our results indicate that acetate supplementation improves growth on propionate by *C. kluyveri* during ethanol-driven chain elongation. The latter strategy could, therefore, be used to enhance production rates in odd-chain elongation processes.

Another relevant finding of our experiments with *C. kluyveri* is that the theoretical ethanol:carboxylic acid stoichiometry of 1.2 does not result in optimal substrate use, at least during odd-chain elongation. According to theory of chain elongation, 1/6th of the substrate (ethanol) is oxidised to acetate for ATP generation and the rest is derived to the reverse β-oxidation pathway [24]. Chain elongation with only acetate does not allow to distinguish between ethanol oxidised to acetate and ethanol used for chain elongation. However, with propionate as electron acceptor, this distinction is possible, since only the even-numbered products (acetate, butyrate, caproate) are the result of ethanol conversion to acetate. Thus, with an ethanol:carboxylic acid ratio of 1.2 (theoretical stoichiometry), the proportion (specificity) of even-chain products in propionate-fed cultures should be 1/6th, or 16.7%. However, in our propionate incubations with this ratio, even-chain carboxylates accounted for 27% of the products (Fig. 3), indicating that more than 1/6th of ethanol was oxidised to acetate. This phenomenon, termed excessive ethanol oxidation, has been described in chain-elongating mixed cultures but attributed to the activity of competing, ethanol-oxidising microorganisms that do not perform chain elongation [49, 50]. Our results are in accordance with those of Candry et al. [51], who showed that *C. kluyveri* also performs excessive ethanol oxidation during odd-chain elongation and that the stoichiometric product output (16.7% even-chain and 83.3% odd-chain) is not achieved with the theoretical E/CA ratio of 1.2. According to that study, the stoichiometric product output is approached with lower ethanol:propionate ratios (e.g., 0.5). It remains a question whether excessive ethanol oxidation also takes place during even-chain elongation or if it is, in fact, a strategy of *C. kluyveri* to deal with propionyl-CoA toxicity and a shortage of acetyl-CoA.

### Application of the synthetic co-culture to upgrade syngas fermentation effluent

Synthetic co-cultures are suitable platforms to upgrade syngas fermentation effluent. Co-cultures such as the one presented in this study are less adaptable than open mixed cultures and are, therefore, not so suited to treat complex organic waste streams (e.g., food waste). However, gasification of feedstocks followed by syngas fermentation results in a rather “clean” effluent: much simpler in composition (mostly ethanol and acetate) and more consistent than organic waste streams, thus easier to handle by monocultures or synthetic co-cultures. Indeed, syngas fermentation effluent, when supplemented with trace metals and vitamins, has been shown to be as good substrate for *C. kluyveri* as synthetic ethanol/acetate mixtures [38].

Contrary to open mixed cultures, synthetic co-cultures can exclude methanogens, which has two advantages when it comes to their use in chain elongation processes. First, the need for methanogenesis inhibitors (e.g., *2*-bromoethanesulfonic acid) is avoided, benefiting both the cost and performance of the process as these chemicals have been shown to lose their effectiveness over time [29, 68]. Second, a neutral, instead of acidic pH can be selected for fermentation (most methanogens grow optimally at pH around neutrality [69]), consequently minimising growth inhibition by accumulation of undissociated carboxylic acids. In addition, multi-species genome-scale metabolic models (GEMs) can be built for synthetic co-cultures to predict phenotypes and find strategies to optimise process performance [70–73]. Recently, we constructed the first GEM of *A. neopropionicum* [53]; in future research, this GEM could be incorporated into, for example, the existing multi-species GEM of the *Clostridium autoethanogenum*–*C. kluyveri* co-culture [71] to evaluate the performance of a synthetic tri-culture applied to syngas fermentation.

An important difference of our study compared to most other works on MCCA production via chain elongation is that we did not provide propionate (or acetate) as electron acceptor, relying only on the endogenous production of SCCAs in the system. In addition, the ethanol loading rates we tested are relatively low compared to those applied in other studies discussed here [49, 50]. Therefore, it is expected that the MCCAs production rates obtained in our study remain relatively low in comparison, but yet prove that the synthetic co-culture has potential to be used as a platform to upgrade dilute ethanol streams to, specifically, OCCAs. Improvement of the *A. neopropionicum*–*C. kluyveri* co-culture could include determining a more optimal pH of operation that could also support higher substrate loading rates. In our bioreactor experiment, increasing the pH from 7 to 7.3 boosted ethanol consumption and chain-elongating activity of C5–C7 carboxylates (phase G, Fig. 6). A pH of 7.3 is closer to the optimum for *A. neopropionicum* [54], and it also contributes to alleviating carboxylic acid toxicity. Among the other strategies, in-line product extraction has been shown to significantly enhance production rates by reducing product inhibition [18, 39, 41, 63]. Biomass retention, either via anaerobic filter reactors or membrane modules, has also been demonstrated to improve production rates [38, 41]. A key finding of our study is that, in line with the work on *C. kluyveri* by Candry and co-authors [51], odd-chain elongation was favoured under ethanol limitation, which can be achieved in controlled chemostat cultivation. Therefore, this approach should be kept in mind when targeting the production of OCCAs via ethanol-driven chain elongation.

## Conclusions

A synthetic co-culture composed of *A. neopropionicum* and *C. kluyveri* was demonstrated to produce valerate and heptanoate from solely ethanol and CO_2_ as substrates. In bioreactor cultivation, the co-culture tolerated concentrations of at least 300 mM ethanol (13.8 g L^−1^) and yielded a maximum of 37% OCCAs. Our results showed that controlling ethanol use is favourable for OCCA production, and that tuning of pH could further boost chain elongation in this co-culture. Moreover, experiments with pure cultures of *C. kluyveri* revealed the negative effects of propionate on its growth, which are reversed when acetate is present. Further work is needed to verify the hypothesis that *C. kluyveri* deals with propionate/propionyl-CoA toxicity by performing excessive ethanol oxidation, which would explain the observed shift towards even-chain products during chain elongation of propionate. In view of the results, we propose that the *A. neopropionicum*–*C. kluyveri* co-culture could be integrated with the syngas fermentation platform to upgrade ethanol to OCCAs. For this, future work should investigate the possibility to establish a tri-culture with an acetogen (one-pot process), or test the co-culture with actual syngas fermentation effluent (two-stage process). In addition, a number of strategies could be explored to improve production rates, such as cell retention, in-line product extraction or co-feeding heterotrophic (waste) substrates. Multi-species GEM modelling is a powerful tool that can also aid in the exploration of capabilities of synthetic co-cultures. Altogether, our work shows the potential of synthetic microbial co-cultures to serve as platforms for innovative biotechnological processes.

## Supplementary Information

**Additional file 1**. Additional data. **Figures S1 and S2**. Cell density and production profiles of batch incubations of *A. neopropionicum* grown on 25–700 mM ethanol. **Figures S3–S4**. Cell density and production profiles of batch incubations of *C. kluyveri* grown on ethanol (120 mM) plus acetate and/or propionate. **Figure S5**. Cell density (OD_600_) of the *A. neopropionicum*–*C. kluyveri* co-culture in ethanol-fed continuous bioreactor.

## Data Availability

All data generated or analysed during this study are included in this published article and its supplementary information file.
