# Supplementary material for: Upgrading dilute ethanol to odd-chain carboxylic acids by a synthetic co-culture of Anaerotignum neopropionicum and Clostridium kluyveri
Source: Biotechnol Biofuels Bioprod. 2023 May 17;16:83. doi: 10.1186/s13068-023-02336-w (PMC10189929; doi:10.1186/s13068-023-02336-w)
Supplement: Supplementary file 1 — Additional file 1. Additional data. Figures S1 and S2. Cell density and production profiles of batch incubations of A. neopropionicum grown on 25–700 mM ethanol. Figures S3–S4. Cell density and production profiles of batch incubations of C. kluyveri grown on ethanol (120 mM) plus acetate and/or propionate. Figure S5. Cell density (OD600) of the A. neopropionicum–C. kluyveri co-culture in ethanol-fed continuous bioreactor. [file 13068_2023_2336_MOESM1_ESM.pdf]

Additional file 1

Upgrading dilute ethanol to odd-chain carboxylic acids by a synthetic co-culture of *Anaerotignum neopropionicum* and *Clostridium kluyveri*

Ivette Parera Olm, Diana Z. Sousa

Figure S1 2
Figure S2 2
Figure S3 3
Figure S4 3
Figure S5 4

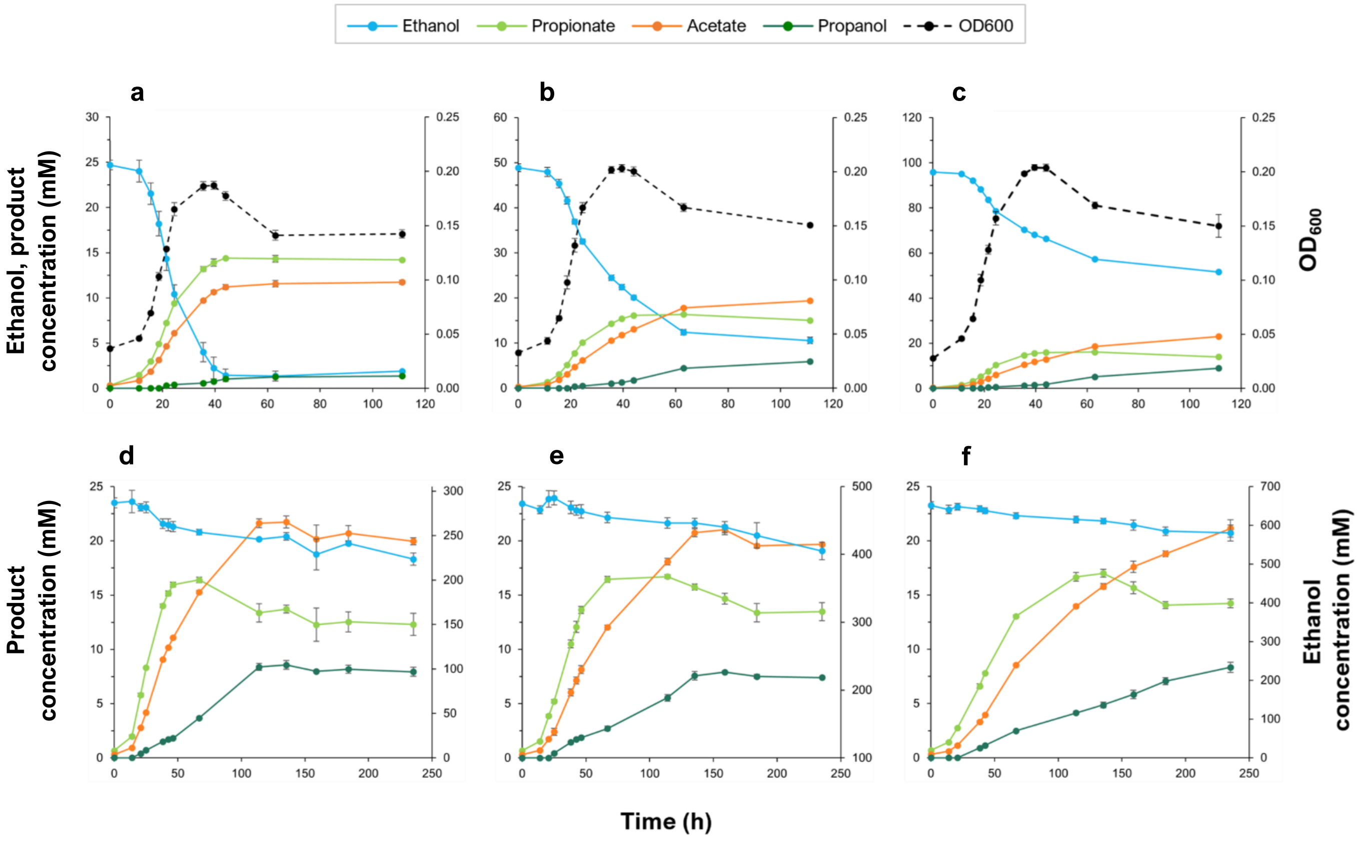

Figure S1. Batch cultivations of *A. neopropionicum* grown on ethanol (25 – 700 mM). **a – c**: Substrate and product concentrations, and cell density (OD_{600}) of incubations containing 25 mM (**a**), 50 mM (**b**) and 100 mM (**c**) ethanol. **d – f**: Substrate and product concentrations of incubations containing 300 mM (**d**), 500 mM (**e**) and 700 mM (**f**) ethanol.

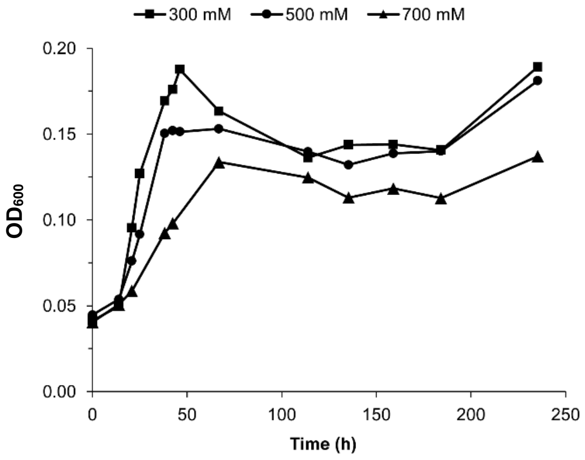

Figure S2. Cell density (measured as OD_{600}) of batch cultures of *A. neopropionicum* grown on 300, 500 and 700 mM ethanol.

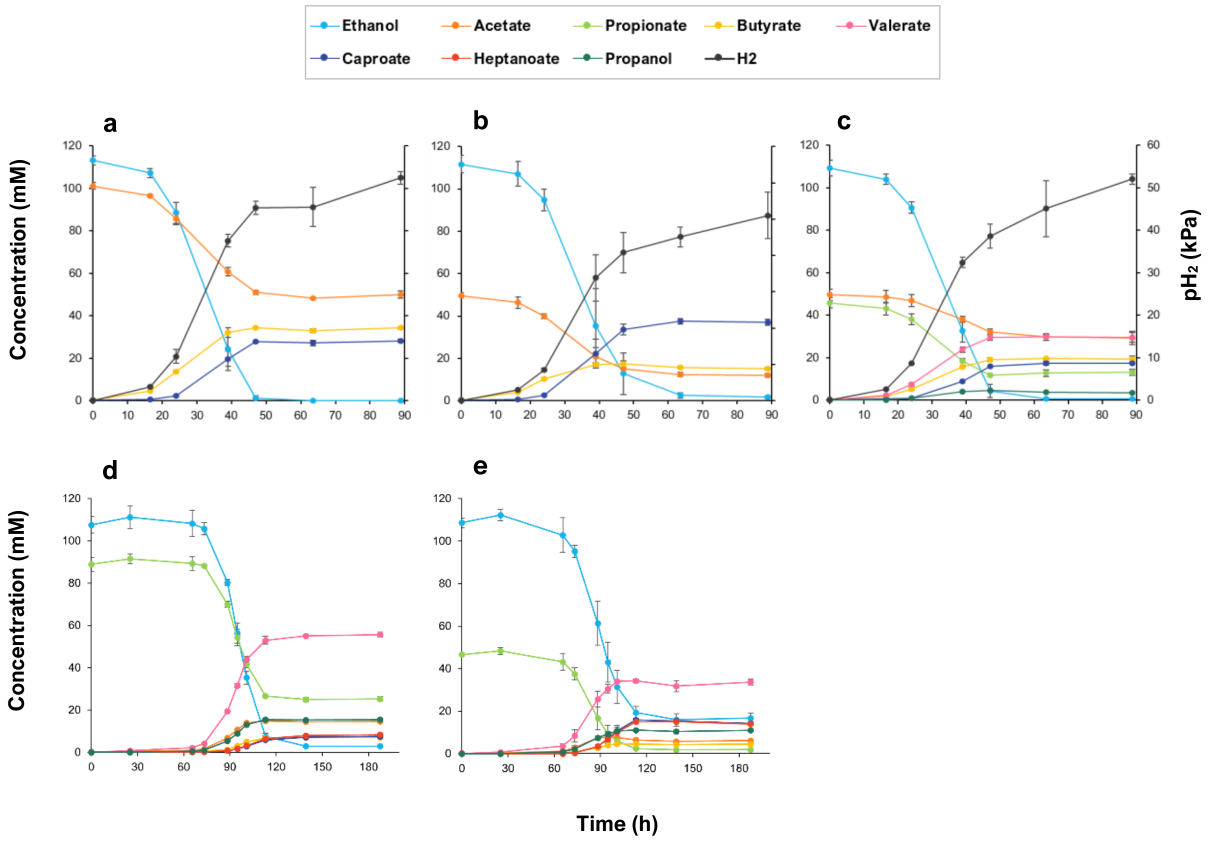

Figure S3. Batch cultivations of *C. kluyveri* grown on ethanol (120 mM) plus acetate and/or propionate. Displayed here are substrate and product concentrations of incubations with: **a**) 100 mM acetate (E/CA = 1.2); **b**) 50 mM acetate (E/CA = 2.4); **c**) 50 mM acetate and 50 mM propionate (E/CA = 1.2); **d**) 100 mM propionate (E/CA = 1.2) and **e**) 50 mM propionate (E/CA = 2.4). Evolution of H_2 was only measured in conditions **a** – **c**; in **d** and **e**, final H_2 pressure was the same as in the other incubations (≈ 50 kPa). Heptanoate was also produced in condition **c** (< 2 mM, not shown).

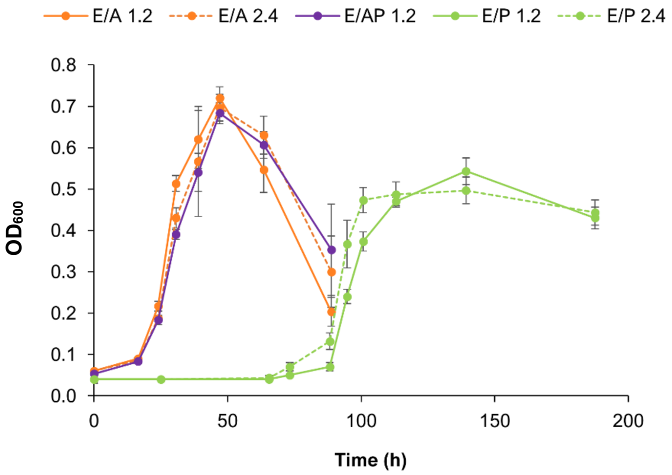

Figure S4. Cell density (measured as OD_{600}) of *C. kluyveri* batch cultures incubated with ethanol (120 mM) and either acetate (E/A), propionate (E/P) or both electron acceptors (E/AP) at the indicated ethanol/carboxylic acid ratio.

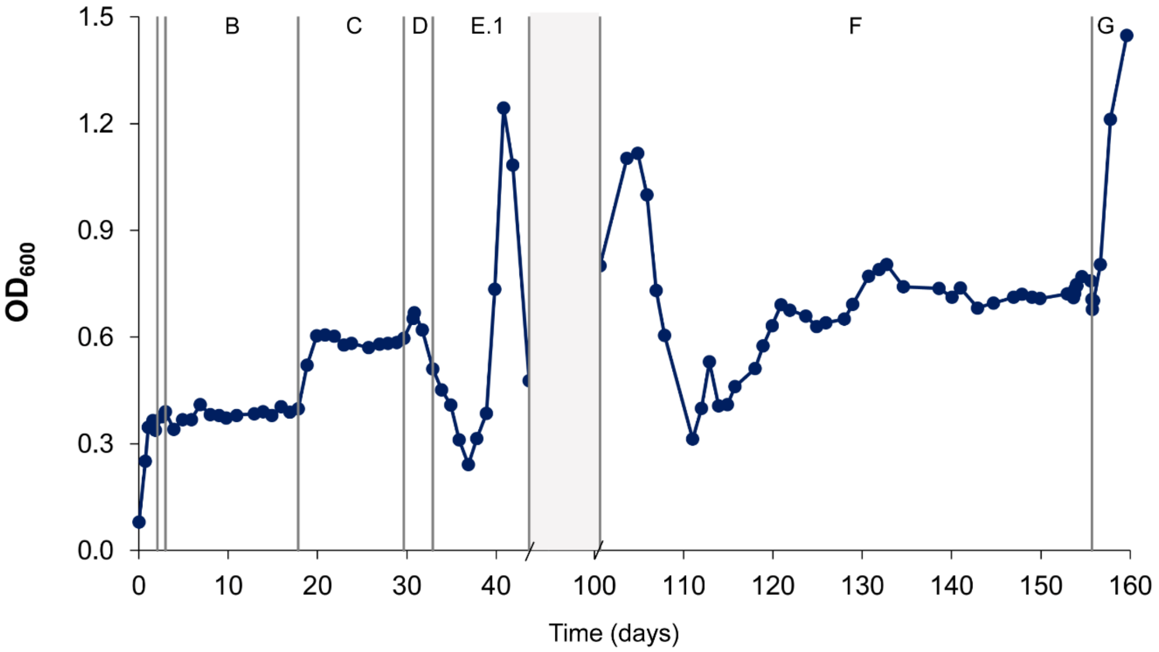

Figure S5. Cell density (OD_{600}) of the *A. neopropionicum* – *C. kluyveri* co-culture in ethanol-fed continuous bioreactor. The different phases of operation (A – F) are indicated (see details in Table 1). The shaded area corresponds to a period where several technical issues occurred and is therefore disregarded.
